# Supplementary material for: Behavioral and environmental determinants of acute diarrhea among under-five children from public health facilities of Siyadebirena Wayu district, north Shoa zone, Amhara regional state, Ethiopia: Unmatched case-control study
Source: PLoS One. 2021 Nov 22;16(11):e0259828. doi: 10.1371/journal.pone.0259828 (PMC8608321; doi:10.1371/journal.pone.0259828)
Supplement: S1 Table — (PDF) [file pone.0259828.s001.pdf]

S1 Table: Distribution of study participants by socio-demographic characteristics, Siyadebirena Wayu District, North Shoa Zone, Amhara region, Ethiopia, 2019

| Characteristic | Participants | |
|---|---|---|
| | Cases (%) | Controls (%) |
| Age group of the mothers in years | | |
| Less than 24 | 12 (11.7) | 17 (8.3) |
| 25-34 years | 49 (47.5) | 108(52.4) |
| Greater than 35 years | 42 (40.8) | 81 (39.3) |
| Maternal education | | |
| Illiterate | 68(66) | 131(63.6) |
| Primary school | 28(27.2) | 54(26.2) |
| Secondary school and above | 7(6.8) | 21(10.2) |
| Occupation of respondent | | |
| Housewife | 86(83.5) | 159(77.2) |
| Civil servant | 6 (5.8) | 22(10.7) |
| Private workers | 11(10.7) | 25(12.1) |
| Occupation of father | | |
| Civil servant | 11 (10.7) | 34 (16.5) |
| Merchant | 16 (15.5) | 23 (11.2 ) |
| Farmer | 74 (71.8) | 143 (69.4) |
| Other* | 2 (2) | 6 (2.9) |
| Birth order of index child | | |
| First | 14 (13. 6) | 29 (14.1) |
| Second | 33 (32) | 66 (32) |
| Third | 24 (23.3) | 41 (19.9) |
| Fourth and above | 32 (31.1) | 70 (34) |

| | | |
|---|---|---|
| Number of family size in household | | |
| Less than five | 65 (63.1) | 128(62.1) |
| Greater than or equal to five | 38 (36.9) | 78 (37.9) |
| Number of under 5 children In the house hold | | |
| One | 75 (72.8) | 152 (73.8) |
| Two | 21(20.4) | 52 (25.2) |
| ≥ 3 | 7(6.8) | 2(1) |
| Age of index child | | |
| 6-11 months | 26(25.2) | 34(16.5) |
| 12-23 months | 38(36.9) | 57(27.7) |
| 24-35 months | 17(16.5) | 41(19.9) |
| ≥36 months | 22(21.4) | 74(35.9) |
| Monthly income of house holds | | |
| ≤ 12 USD | 46(44.7) | 62(30.1) |
| Between 13 and 23 USD | 32(31.1) | 20(3.7) |
| ≥ 24 USD | 25(24.2) | 124(60.2) |
