# Supplementary material for: Behavioral and environmental determinants of acute diarrhea among under-five children from public health facilities of Siyadebirena Wayu district, north Shoa zone, Amhara regional state, Ethiopia: Unmatched case-control study
Source: PLoS One. 2021 Nov 22;16(11):e0259828. doi: 10.1371/journal.pone.0259828 (PMC8608321; doi:10.1371/journal.pone.0259828)
Supplement: S2 Table — (PDF) [file pone.0259828.s002.pdf]

S2 Table: Distribution of environmental conditions of study participants in Siyadebirena Wayu District, North Shoa Zone, Amhara region, Ethiopia, 2019.

Characteristic	Participants	
	Cases (%) (n=103)	Controls (%) (n=206)
Water source		
Unprotected	13(12.6)	4(1.9)
Protected	90(87.4)	202(98.1)
Treat drinking water at home		
Yes	17(16.5)	150(72.8)
No	86(83.5)	56(27.2)
Availability of latrine		
Yes	78(75.7)	166(80.6)
No	25(24.3)	40(19.4)
Separate house for domestic animals		
Yes	89(86.4)	199(96.6)
No	14(13.6)	7(3.4)
Time to fetch drinking water (round trip)		
<15 minutes	60(58.3)	119(57.8)
15-30 minutes	42(40.8)	81(39.3)
>30 minutes	1(0.9)	6(2.9)
Disposes infant feces		
Inside the latrine	17(16.5)	153(74.3)
Outside the latrine	86(83.5)	53(25.7)
Human feces are seen around the house		
Yes	40(51.3)	40(24.1)
No	38(48.7)	126(75.9)
Human feces are seen around the pit hole		
Yes	41(52.6)	28(16.9)
No	37(47.4)	138(83.1)
