# Supplementary material for: Behavioral and environmental determinants of acute diarrhea among under-five children from public health facilities of Siyadebirena Wayu district, north Shoa zone, Amhara regional state, Ethiopia: Unmatched case-control study
Source: PLoS One. 2021 Nov 22;16(11):e0259828. doi: 10.1371/journal.pone.0259828 (PMC8608321; doi:10.1371/journal.pone.0259828)
Supplement: S3 Table — (PDF) [file pone.0259828.s003.pdf]

S3 Table: Behavioral factors of study participants in relation to acute diarrhea, Siyadebirena Wayu District, North Shoa Zone, Amhara region, Ethiopia, 2019

Characteristic	Participants	
	Cases (%) (n=103)	Controls (%) (n=206)
Hand-washing		
Yes	79(76.7)	201(97.6)
No	24(23.3)	5(2.4)
Hand-washing materials (n=280)		
With water only	54(68.4)	37(18.4)
With soap	25(31.6)	164(81..6)
Use left-over food		
Yes	58(56.3)	49(23.8)
No	45(43.7)	157(76.2)
Used covered water storage		
Yes	97(94.2)	192(93.2)
No	6(5.8)	14(6.8)
Means of drawing drinking water		
Pouring	77(74.8)	191(92.7)
Dipping	26(25.2)	15(7.3)
Preparing child food separately		
Yes	86(83.5)	193(93.7)
No	17(16.5)	13(6.3)
Maternal history of recent diarrhea		
Yes	29(28.2)	25(12.1)
No	74(71.8)	181(87.9)
Measles immunization		
Yes	69(67)	178(86.4)
No	34(33)	28(13.6)
Rota Virus immunization		
Yes	76(73.8)	191 (92.7)
No	27(26.2)	15(7.3)
Vitamin "A" supplement		
Yes	41 (39.8)	33 (14.1)
No	62 (60.2)	177 (85.9)
Hand washing practices		
Poor	82(79.6)	120(58.3)
Good	21(20.4)	86(41.7)
Knowledge of major risk factors		
Good	13(12.6)	129(62.6)
Poor	90(87.4)	77(37.4)
