# Supplementary material for: Behavioral and environmental determinants of acute diarrhea among under-five children from public health facilities of Siyadebirena Wayu district, north Shoa zone, Amhara regional state, Ethiopia: Unmatched case-control study
Source: PLoS One. 2021 Nov 22;16(11):e0259828. doi: 10.1371/journal.pone.0259828 (PMC8608321; doi:10.1371/journal.pone.0259828)
Supplement: S1 Annex — (PDF) [file pone.0259828.s004.pdf]

English Version Questionnaire

Debre Berhan University, Institute of Medicine and Health Science, College of Health Sciences Department of Public Health

Questionnaire Prepared to Assess the Determinants of Acute Diarrhea among 6-59 months old Children in Public Health Facilities Siyadebirena Wayu district, 2019

IDENTIFICATION

001. ID numbers of the Questionnaire: ____________

002. Address: Rural: _________ Urban: ___________

003. Kebele: __________

PART I. SOCIODEMOGRAPHC CONDITIONS

No	Questions	Options (responses)
Q101	Number of persons in the house	---------------------- in number
Q102	Number of under-five children in house	--------------------- in number
Q103	Age of the index child	-------------------in months
Q104	Sex of the index child	1. Male 2. Female
Q105	Where was your child born?	1. Health institution 2. Home
Q106	Birth order of the child	1. First 2. Second 3. Third 4. Fourth & above
Q107	Relation of the respondent to the child	1. Mother 2. Caretaker
Q108	Age of the mother/caretaker	__________ Years
Q109	Marital status of the mother/caretaker	1. Married 2. Divorced<br>3. Single 4. Widowed
Q110	Religion of parents/caretaker	1. Orthodox 2.Protestant<br>3. Muslim 4. Catholic<br>5. Other (specify)
Q111	Ethnic group of parents/caretakers	1. Amhara 2. Oromo<br>3.Tigrai 4.Others
Q112	Educational level of mother/caretaker	1. Unable to read and write

		2. Able to read and write<br>3. Primary school<br>4. Secondary school and above
Q113	Occupation of the mother/caretaker	1 Housewife<br>2. Government employee<br>3. Private worker<br>4. Other (specify)_______________
Q114	Age of the child's father	__________Years
Q115	Educational level of the father	1. Unable to read and write<br>2. Able to read and write<br>3. Primary school<br>4. Secondary school and above
Q116	Occupation of the father	1. Government employee<br>2. Merchant<br>3. Farmer<br>4. Other (specify
Q117	Average monthly income of the family	------------------- in USD
Q118	Does the family own farmland	1. Yes 2. No
Q119	Does the family have TV	1. Yes 2. No
Q120	Does the family have mobile phone	1. Yes 2. No
Q121	Does the family have bank account	1. Yes 2. No
Q122	Does the family have radio	1. Yes 2. No
Q123	Does the family have electric light	1. Yes 2. No
Q124	Does the family have oxen	1. Yes 2. No
Q125	Does the family have cow	1. Yes 2. No
Q126	Does the family have sheep	1. Yes 2. No
Q127	Does the family have goat	1. Yes 2. No
Q128	Does the family have hens	1. Yes 2. No
Q129	Does the family have donkey	1. Yes 2. No
Q130	Does the family mule/horse	1. Yes 2. No

Q131	Does the family have a house	1. Yes 2. No
Q132	Does the family produce cash crop	1.teff<br><br>2.wheat<br><br>3. Barley<br><br>4. Other cereals

PART II. ENVIRONMENTAL HEALTH CONDITIONS

Q201	Type of floor material of the living house	1. Mud 3. Cement<br><br>2. Wood 4. Other (specify)
Q202	Type of roof material of the living house	1. Thatched<br><br>2. Corrugated iron sheet<br><br>3. Other (specify)
Q203	Do animals live in the same house where the members of the family live?	1. Yes 2. No
Q204	Number of rooms in the house	_________
Q205	Is latrine available?	1. Yes<br><br>2. No (If No, skip to Q209)
Q206	Ownership of the latrine	1. Privately owned<br><br>2. Shared with neighbors
Q207	Is feces seen around the pit-hole (or on the floor)?	1. Yes 2. No
Q208	Is feces seen around the house (or in the compound)?	1. Yes 2. No
Q209	If the family has no latrine, where do you dispose human waste?	1. Open field<br><br>2. Other (specify)
Q210	Where dispose child feces	1.in latrine 2.not in latrine
Q211	How do you dispose refuse?	1. Pit 3. Open field<br><br>2. Burning 4. Garbage can<br><br>5.Other

Q212	From where do you get water for drinking?	1. Pipe<br><br>2. Protected well/spring<br><br>3. Unprotected well/spring<br><br>4. River<br><br>5. Other (specify)
Q213	Distance from the house to the water source	__________ Minutes
Q214	Type of collection container	1. Pot 4. Jerry can<br><br>2. Plastic bucket 5. Other<br><br>3. Iron bucket
Q215	How did you transport the collected drinking water to the house yesterday?	1. In a covered container<br><br>2. In an uncovered container<br><br>3. Other (specify)
Q216	Capacity of the container, which you used to collect drinking water yesterday?	_______ Liters
Q217	How many times did you collect water for drinking Yesterday?	---------------times
Q218	Does the drinking-water storage container have a cover?	1. Yes 2. No
Q219	Is there a separate can for taking drinking water from the storage container?	1. Yes 2. No
Q220	How do you take water from the drinking water storage container?	1. Pouring<br><br>2. Dipping

PART III. BEHAVIORAL ASPECTS

Q301	Does the child take other food than breast milk?	1. Yes 2. No
Q302	Do you separately prepare food for the child, using a separate material?	1. Yes 2. No

Q303	What food/fluid is the child mostly receiving (if the child is not on exclusive breastfeeding)?	1. Cow's milk 4. Adults' food<br><br>2. Powder milk 5. Other
Q304	What do you use to feed the child?	1. Hand 4. Bottle<br><br>2. Cup and spoon 5. Other<br><br>3. Cup
Q305	Are you give leftover food to your child	1. Yes 2. No
Q306	Mother/caretaker have history of diarrhea in the past two weeks?	1. Yes 2. No
Q307	Have you ever breast-fed your child?	1. Yes 2. No
Q308	For how long did you breastfed your child?	__________Months
Q309	What is his/her current breastfeeding status?	1. Exclusive breastfeeding<br><br>2. Partial breastfeeding<br><br>3. Not breastfeeding
Q310	At what age the child started supplementary /weaning food?	________ Months
Q311	Did the child receive Rota vaccination	1. Yes, (by the response of the respondent)<br><br>2. Yes, (by checking the card)<br><br>3. No
Q312	Did the child receive measles vaccination?	1. Yes, (by the response of the respondent)<br><br>2. Yes, (by checking the card)<br><br>3. No
Q313	Did the child receive Vit A supplementation	1.Yes 2.No
Q314	Do you wash your hand	1. Yes 2. No
Q315	When do you wash your hands? (more than	1.Before eating

		2.After eating<br><br>3.After toilet visit<br><br>4.After cleaning the child<br><br>5.Before preparing food
Q316	Cleansing material for hand washing?	1. Soap 2. Water only
Q317	Do your child have diarrhea today?	1. Yes 2. No
Q318	For how long the diarrhea last?	-----------------days
Q319	If the child has diarrhea today, how many times a day<br><br>He/she passes stool?	1. Three times<br><br>2. More than three times<br><br>3. Don't know
Q320	The type of diarrhea that the child had	1. Watery<br><br>2. Blood and mucus
Q321	What actions do you take to treat/stop the diarrhea?	1. Take him/her to health<br><br>institution<br><br>2. Take him/her to traditional<br><br>healer<br><br>3. Increase feeding<br><br>4. Give him/her ORS<br><br>5. Give him/her cereal based fluids<br><br>6. Stop/decrease feeding<br><br>7. Homemade treatment<br><br>8. Other (specify)
Q322	Do you know that flies can transmit diseases?	1. Yes 2. No
Q323	If “Yes”, can you tell me the name of the diseases?	1. Diarrhea

		2. Typhoid fever<br><br>3. Cholera<br><br>4. Trachoma<br><br>5. Do not know the names<br><br>6. Other (specify
Q324	Do you know that excreta of children can be a cause of diseases?	1. Yes 2. No
Q325	Do you know the list major risk factor of acute diarrhea	1. Non vaccinated for Rota virus<br><br>2.Lack of early initiation and exclusive Breast feeding<br><br>3.Using unsafe water for drinking<br><br>4.Lack of hand washing practice<br><br>5.Eating improperly handled food<br><br>6.lack of latrines facility

Date of interview ______________________________

Name of the interviewer _________________________

Signature ______________________________________

QUESTIONNAIRE- AMHARIC VERSION

በደብረብርሃን ዩንቨርስቲ ጤና ሳይንስ ኮሌጅ የህብረተሰብ ጤና ት/ክፍል

ከ6-59 ወር እድሜ ባሉ ህፃናት ላይ የሚከተለውን ድንገተኛ የተቅማጥ በሽታ ወሳኝ ምክኒያቶችን ለማወቅ የተዘጋጀ መጠይቅ

<u>ቃለመጠይቅን ከመጀመርህ በፊት የሚከተሉትን ነጥቦች አስታውስ</u>

ለጥናቱ የተመረጠው ህፃን እናት መሆኑዋን ቃለ መጠይቅ ከመጀመርህ በፊት አረጋግጥ፡፡ለቃለ መጠይቅ ፈቃድ መጠየቅ ይኖርብሃል፡፡ፈቃድ ሳታገኝ በፍፁም መጠየቅ የለብህም፡፡

ስምህን በመናገር ራስህን አስተዋውቅ :የጥናቱን አላማና ለጥናቱ ለምን ና እንዴት እንደተመረጡ ለተሳታፊው አስረዳ

የህፃኑን እናት /አሳዳጊ የጥናቱን አላማ " የስምምነት መጠየቂያ" ከሚለው በታች የተፃፈውን በማንበብ ለቃለመጠይቅ ፈቃደኛ መሆኑን ጠይቅ: ቃለምልልሱን ከጨረስክ በኃላ ቃለምልልሱን እንደጨረስክ ተናግረህ ግለሰቡን አመስግነህ ወደሚቀጥለው እለፍ

የስምምነት መጠየቂያ

ስሜ -------------------ይባላል፡፡ የምሰራው ደነባ ጤና ጣቢያ/ደነባ ሆስፒታል ነው፡፡ይህ መጠይቅ በህፃናት ላይ የሚከሰተውን ድንገተኛ ተቅማጥ የሚያመጡ ዋና ዋና ምክንያቶች ለማወቅ የተዘጋጀ መጠይቅ ነው፡፡መጠይቁ የሚሞላው በቃለ ምልልሱ ሲሆን የሚሰጡት ምላሽ ለማንም ግለሰብ ወይም ድርጅት ተላልፎ የማይሰጥና ሚስጥራዊነቱም የተጠበቀ ይሆናል፡፡ጥናቱ ዉጤታማ ሊሆን የሚችለው እርስዎ በሚሰጡት ትክክለኛ መልስ በመሆኑ ጥያቄዎቹን በትክክል እንዲመልሱልን ፈቃደኝነትዎትን በትህትና እንጠይቃለን፡፡

ቃለምልልሱን ለማድረግ ተስማምተዋል ? አዎ ተስማምቻለሁ-----አልተስማማሁም----------(ወደሚቀጥለው እለፍ)

መለያ

001፡ የመጠየቂያ

መለያ ቁጥር

002፡ አድራሻ :ገጠር-------ከተማ:-----------

003፡ ቀበሌ**:** ______

<u>ማህበራዊ ና ኢኮኖሚያዊ ሁኔታዎች</u>

ተ.ቁ	ጥያቄዎች ና ማጣሪያዎች	አማራጭ መልሶች
Q101	የቤተሰብ ብዛት	-------------------በቁጥር
Q102	እድሜያቸው ከአምስት አመት በታች ያሉ ህፃናት ብዛት	------------------በቁጥር
Q103	የተመረጥው ህፃኑ እድሜ	------------------በወር
Q104	የተመረጠው ህፃኑ ፆታ	1.ወንድ 2. ሴት
Q105	ህፃኑ የተወለደበት ቦታ	1.በጤና ተቋም 2. በቤት ዉስጥ
Q106	ህፃኑ ለእናትየው ስንተኛ ልጅ ነው	1.አንደኛ 2. ሁለተኛ 3. ሶስተኛ 4. አራተኛ ና ከዚያ በላይ
Q107	መላሹ ከ ህፃኑ ጋ ያለው ግንኙነት	1.እናት 2.ሌላ አሳዳጊ
Q108	የህፃኑ እናት/አሳዳጊ እድሜ	--------አመት
Q109	የህፃኑ እናት/አሳዳጊ የጋብቻ ሁኔታ	1.ያገባች 2. የተፋታች 3. ያላገባች 4. ባል በሞት የተለያት

Q110	የህፃኑ ወላጆች/ አሳዳጊዎቸ ሃይማኖት ሁኔታ	1.ክርስቲያን 2. ሙስሊም<br><br>3.ፕሮቴስታንት 4. ሌላ ካለ ይገለፅ
Q111	የህፃኑ ወላጆች/ አሳዳጊዎቸ ዘር/ጎሳ	1.አማራ 2.ኦሮሞ 3.ትግሬ 4.ሌላ ካለ ይገለፅ
Q112	የህፃኑ እናት/አሳዳጊ የትምህርት ደረጃ	1.መደበኛ ትምህርት 2. መጻፍና ማንበብ 3.ማንበብ ብቻ 4. ምንም ያልተማረ
Q113	የህፃኑ እናት/አሳዳጊ የስራ ሁኔታ	1.የቤት እመቤት 2. የመንግስት ሰራተኛ 3. ገቢ የሚያስገኝ የግል ስራ 4. ሌላ ይገለፅ
Q114	የህፃኑ አባት እድሜ	--------አመት
Q115	የህፃኑ አባት የትምህርት ደረጃ	1.መደበኛ ትምህርት 2. መጻፍና ማንበብ 3.ማንበብ ብቻ 4. ምንም ያልተማረ
Q116	የህፃኑ አባት የስራ ሁኔታ	1. የመንግስት ሰራተኛ 2. ገበሬ4. ነጋዴ 3. ሰራ የለውም 4. ሌላ ይገለፅ
Q117	የቤተሰብ ገቢ ደረጃ	--------------------ብር
Q118	ቤተሰቡ የእርሻ መሬት አለው	1.አለው 2. የለውም
Q119	ቤተሰቡ ቲቪ አለው	1.አለው 2. የለውም
Q120	በቤተሰቡ ሞባይል አለው	1.አለው 2. የለውም
Q121	ቤተሰቡ ባንክ አካዉንት አለው	1.አለው 2. የለውም
Q122	ቤተሰቡ ሬድዎ አለው	1.አለው 2. የለውም
Q123	ቤተሰቡ ኤሌክትሪክ መብራት አለው	1.አለው 2. የለውም
Q124	ቤተሰቡ በሬዎች አሉት	1.አለው 2. የለውም
Q125	ቤተሰቡ ላሞች አሉት	1.አለው 2. የለውም
Q126	ቤተሰቡ በጎች አሉት	1.አለው 2. የለውም
Q127	ቤተሰቡ ፍየሎች አሉት	1.አለው 2. የለውም
Q128	ቤተሰቡ ዶሮዎች አሉት	1.አለው 2. የለውም
Q129	ቤተሰቡ አህያ አለው	1.አለው 2. የለውም
Q130	ቤተሰቡ ፈረስ አለው	1.አለው 2. የለውም
Q131	ቤተሰቡ መኖሪያ ቤት አለው	1.አለው 2. የለውም
Q132	ቤተሰቡ በዚህ አመት ለሽያጭ የሚዉሉ አትክልቶችን ያበቅላል(ጤፍ ገብስ ስንዴ ና ሌሎች) ይጨምራል	1. ጤፍ 2. ገብስ 3. ስንዴ 4. ሌሎች

ክፍል ሁለት: የአካባቢ ጤና ሁኔታ

Q201	የመኖሪያ ቤት ወለሉ ሁኔታ	1.አፈር 2.እንጨት (ጣዉላ) 3.ሲሚንቶ 4. ሌላ ይገለፅ
Q202	የመኖሪያ ቤት ጣሪያ የተሰራበት ሁኔታ	1.የሳር ክዳን 2. የቆርቆሮ ክዳን 3. ሌላ ይገለፅ
Q203	እንሰሳት በቤተ ዉስጥ ከሰዎች ጋር አብረው ይኖራሉ	1.አዎ 2. አይኖሩም
Q204	በቤተ ዉስጥ ያሉት ክፍሎች ብዛት	----- ---------------------------
Q205	ቤተሰቡ መፀዳጃ ቤት	1.አለው 2.የለውም ((መልሱ የለዉም ከሆነ ወደቀ208 እለፍ)
Q206	የ መፀዳጃ ቤት የባለቤትነት ሁኔታ	1.የግል 2. የጋራ

Q207	በ መፀዳጃ ቤት አካባቢ የሰው አይነምድር ይታያል	1.አዎ 2. አይታይም
Q208	መፀዳጃ ቤት ከሌለ ቤተሰቡ የት ይጠቀማል	1.በየሜዳው 2.ሌላ ይገለፅ
Q209	የህፃኑ አይነ -ምድር የት ያስወግዱታል	1.ሽንት ቤት 2. ከሽንት ቤት ዉጭ
Q210	ደረቅ ቆሻሻ እንዴት ያስወግዳሉ	1.በጉድገዋድ ዉስጥ 2.በማቃጠል 3. በየሜዳው 4.በእቃ አጠራቅመው ሌላ ቦታ ይደፋሉ 5. ሌላ ይገለፅ
Q211	የመጠጥ ዉሃ ከየት ያገኛሉ	1.ከቧንቧ 2.ከተጠበቀ የዉሃ ጉድጓድ/ምንጭ 3. .ካልተጠበቀ የዉሃ ጉድጓድ/ምንጭ 4.ከወንዝ 5. ሌላ ካለ ይገለፅ
Q212	ዉሃ በሚቀዳበት ና በቤቱ መካከል ያለው የደርሶ መልስ ርቀት	---------ደቂቃ
Q213	በትናትናው እለት ለመጠጥ የሚሆነውን ውሃ የቀዱበት እቃ ምን አይነት ነበር	1.እንስራ 2.ባልዲ ፕላስቲክ 3.ባልዲ ብረት 4.ጀሪካን 5. ሌላ ይገለፅ
Q214	በትናትናው እለት ለመጠጥ የሚሆነውን ውሃ ከቀዱ በኋላ ወደቤት የወሰዱበት እንዴት ነበር	1.ክዳን ባለው ዕቃ 2. ክዳን በሌለው እቃ 3. በቅጠል በተከደነ እንስራ 4. ሌላ ካለ ይገለፅ
Q215	በትናትናው እለት ለመጠጥ የሚሆነውን ውሃ ቀዱበት ዕቃ የሚይዘው የውሃ መጠን	--------------ሊትር
Q216	በትናትናው እለት ስንት ጊዜ ውሃ ቀዱ/አመላለሱ	-----------ጊዜ
Q217	የመጠጥ ውሃ ማጠራቀሚያው/ ማምጫው እቃ ክዳን አለው	1.አለው 2. የለውም
Q218	ተለይቶ የተቀመጠ የውሃ መቅጃ እቃ አለ	1.አለ 2. የለም
Q219	የመጠጥ ውሃ ማጠራቀሚያው/ ማምጫው እቃ ክዳን አለው	1.አለው 2. የለውም

ክፍል 3 ፡የህፃናት አያያዝ ልምድ ና እውቀት

Q301	ህፃኑ ከእናት ጡት ሌላ ምግብ ይወስዳል	1.አዎ<br>2. የጡት ወተት ብቻ ነው የሚመገበው 306 እለፍ
Q302	ህፃኑ ለብቻው ምግብ ያዘጋጁለታል	1.አዎ<br>2. አላዘጋጅም
Q303	ህፃኑ ከጡት ወተት ሌላ ምግብ የሚወስድ ከሆነ ምን አይነት ምግብ ይወስዳል(ከአንድ በላይ መልስ ሊኖረው ይችላል)	1.የላማ ወተት<br>2. የዱቄት ወተት<br>3. ከተፈጨ ጥራጥሬ የተዘጋጀ ምግብ<br>4.ለአዋቂ የተዘጋጀ ምግብ<br>5. ሌላ ይገለፅ
Q304	ህፃኑን በምን ይመግቡታል	1.በእጄ

		2. በስኒና በማንኪያ<br><br>3. በኩባያ 4. በጡጦ<br><br>4. ሌላ ካለ ይገለፅ
Q305	የተረፈ ምግብ ለልጅዎ ይሰጡታል	1. አዎ<br>2. 2. አልሰጥም
Q306	እናትየዋ/ አሳዳጊዋ ባለፈው ሁለት ሳምንት ዉስጥ ተቅማጥ ይዟቸው ነበር	1.አዎ<br><br>2. አልያዘኝም
Q307	ህፃኑ ለምን ያክል ወር ጠባ	-----ወር
Q308	ህፃኑ ተጨማሪ ምግብ የጀመረበት ጊዜ	----ወር
Q309	ህፃኑ ሮታ ክትባት ተከትቧል	1.አዎ ከመላሹ የተገኘ<br><br>2.አዎ ከመዝገብ የተገኘ<br><br>3.አልተከተበም
Q310	ህፃኑ ሚዝል ክትባት ተከትቧል	1.አዎ ከመላሹ የተገኘ<br><br>2.አዎ ከመዝገብ የተገኘ<br><br>3.አልተከተበም
Q311	ህፃኑ ቫይታሚን ኤእንክብል ወስደዋል	1.አዎ 2. አልወሰደም
Q312	እጅዎን ይታጠባሉ	1.አዎ 2. አልታጠብም
Q313	እጅዎን የሚታጠቡት ምቼ ነው	1 ከምግብ በፊት<br><br>2.ከምግብ በኃላ<br><br>3.ከመፀዳጃ ቤት መልስ<br><br>4. ህፃኑን ካፀዳዳሁ በኃላ<br><br>5. ምግብ ከመዘጋጀቱ በፊት
Q314	እጅዎን ለመታጠብ ምን ይጠቀማሉ	1. ዉሃ ብቻ<br><br>2.ሳሙና ወይም አመድ
Q315	በአሁኑ ጊዜ ህፃኑ ተቅማጥ አለው	1.አዎ መላሹ የተገኘ<br><br>2. በባለሙያ የተረጋገጠ

		3. የለበትም
Q316	ህፃኑ ተቅማጡ ስንት ጊዜ ቆየበት	---------------------------ቀን
Q317	በቀን ዉስጥ ስንት ጊዜ ያስቀምጠዋል	1. ሶስትጊዜ<br>2. ከሶስትጊዜበላይ<br>3. አላውቅም
Q318	ምን አይነት ተቅማጥ ነው ፡፡	1.እንደዉሃ የቀጠነ<br>2. ደም ና ዉሃ የተቀለቀለበት<br>3. ሌላ ይገለፅ
Q319	ህፃኑ ተዛማጅ በሽታ አለው ፡፡	1.የሳምባ ምች/pnumonia/<br>2. የምግብ እጥረት/SAM/<br>3. ሌላ ይገለፅ
Q320	ዝንብ በሽታ ልታስተላልፍ እንደምትችል ያውቃሉ	1.አውቃለሁ<br>2. አላውቅም መልሱ አላዉቅም ከሆን ወደ ቀ310 እለፍ
Q321	የ319 መልሱ አውቃለሁ ከሆነ ልታስተላልፍ ይምትችለውን የበሽታ ስም ሊነግሩኝ ይችላሉ	1.ተቅማጥ<br>2. ታይፎይድ<br>3. ኮሌራ<br>4.አይነ ማዝ/ትራኮማ<br>5. ስማቸውን አላዉቅም 6. ሌላ (ይገለፅ)
Q322	ተቅማጡን ለማቆም ለህፃኑ ምን አድርገዉለታል;	ወደጤናድርጅትወስጀዋሁ<br>ወደባህል ህክምና ወስጀዋለሁ<br>ወትሮ ከሚበላው ምግብ ተጨማሪምግብ እሰጠዋለሁ<br>ኦአርኤስ/ORS/ እሰጠዋለሁ<br>ከተፈጨ ጥራጥሬ የተዘጋጀ ፈሳሽ እሰጠዋለሁ<br>ወትሮ ከምሰጠው ምግብ ያነሰ እሰጠዋለሁሁ<br>በግል መድሃኒት ገዝቼ ሰጥቼዋለሁ<br>ሌላ ይገለፅ

Q323	የተቅማጥ ዋና ዋና መንስኤዎች ያዉቃሉ	1.ሮት ክትባት አለመከተብ<br><br>2. የእናት ጡት ዘግይቶ ማስጀመር እና ለ 6 ወር ጡት ብቻ አለመስጠት<br><br>3.የተበከለ ዉሃ ለመጠጥ መጠቀም<br><br>4. እጅ ዉሃ መታጠብ ልምድ አለመኖር<br><br>5. በአግባቡ ንፅህናው ያልተጠበቀ ምግብ መጠቀም<br><br>6. ሽንት ቤት አለመኖር እና አለመጠቀም

መጠይቁ የተደረገበት ቀን -------------------------መጠይቁን የሞላው ባለሙያ ---------------------ፊርማ-----------
