# Supplementary material for: Behavioral and environmental determinants of acute diarrhea among under-five children from public health facilities of Siyadebirena Wayu district, north Shoa zone, Amhara regional state, Ethiopia: Unmatched case-control study
Source: PLoS One. 2021 Nov 22;16(11):e0259828. doi: 10.1371/journal.pone.0259828 (PMC8608321; doi:10.1371/journal.pone.0259828)
Supplement: Attachment — Submitted filename: 7. My comments.docx [file pone.0259828.s006.docx]

**Date 19/5/2021**

**Comments for authors or editors:**

Thank you for asking to review your paper titled “Behavioral and Environmental Determinants of Acute Diarrhea among Under-five Children from Public Health Facilities of Siyadebirena Wayu District, North Shoa Zone, Amhara Regional State, Ethiopia: Unmatched Case-control Study.”

**General critique**

To me, the paper is important to enhance and to recall the existing body of knowledge about behavioral and environmental determinants of acute diarrhea among Under-five Children. However, I have several concerns that need to be address before I can say that it is ready for publication. Besides, I will also suggest some editorial changes.

**Abstract**

The abstract is relatively good.

Page 2 line 33 “Methods: “Facility-based unmatched case-control study design was employed from March 12, 34 2019, to May 12, 2019.”

Better to be *“****Facility-based unmatched case-control study was conducted from March 12, 2019, to May 12, 2019.”***

Page 2 line 37 “The binary logistic regression model was employed to evaluate the independent effect of the predictor variables on acute childhood diarrhea”.

Better to be “***To analysis the data, binary logistic regression and multivariable logistic regression analysis was conducted***.”

**Introduction**

With the exception of some repeated and an attractive sentences, the introduction part of the paper is sound. Moreover, this part lacks essential references, particularly for those sentences that states figure like in page (P) 3, line (L) 76-77 which states “Annually, it is estimated that 1.3 million deaths are associated with diarrheal diseases with the most occurring in resource-limited countries” and P3, L 67-68 which states “Acute diarrhea is a major public health problem in the world. Next to pneumonia, it is the leading cause of death in children under five years old.” And soon.

P3,L 58-60 which states “It is caused by bacterial, viral, and parasitic organisms and is usually a symptom of gastrointestinal infection **which can be caused by a variety of bacterial, viral, and parasitic organisms. Please change it into”** It is caused by bacterial, viral, and parasitic organisms and is usually causes gastrointestinal infection.”

P3 L62, please add the word “practice” after the word “sanitation”

P3 L82, delete the word “the”

P4 L88 add (,) after the word” revealed that”

**Methods**

The method part of the paper is relatively sequential, logical and well-constructed except the analyzing method.

***Study area:***

Although it is not supported by references, the “topic of study area” provided good information to readers.

***Data collection tools and methods***

P8 L201, “Data was collected by a nurse who works at the under-five OPD/inpatient pediatrics ward of the health center and hospital.” Do you mean the data was collected by 1 nurse? Did you provided training for 1 data collector? I do not think so. See it.

P8 L208, delete the word “the” and replace it with “A”

**Data Processing and Analysis**

Here I have a great concerned.

P8 L235-238, stated that “To avoid an excessive number of variables and unstable estimates in the subsequent model, only variables with a p-value < 0.20 were kept in the analyses. Independent variables which result in a p-value less than 0.20[15] in an unadjusted model are candidates to be considered for the final multivariable model.” I am not satisfying with the reason that the authors provided. Rather, in my opinion, this reason caused to reject may core, known and scientifically assured determinant variables that affect positively or negatively to diarrheal disease in the analysis like Rotavirus vaccine, type of sources of drinking-water etc..

Scientifically, in binary logistic regression analysis, a variable which has a P-value < 0.05 must be included in the multivariable analysis. And based on the result of the multivariable analysis any one can discuss and conclude his/her findings. However, the authors could not follow this idea.

**Results**

This part has many grammatical problems and figure which are not present in the respected tables. In short, it is written in a poor manner.

P10 L252 delete (’) which is located after the word “respondents.”

P10 L255 states “ Forty-nine **(47.6 %)”** which is wrong.

P10 L 256 – 257, stated “Regarding the **age of children,** the average was 32.31 257 (±5.6) years for cases and 32.18 (±5.6) for controls. Can we call children whose age 32.3 and 32. 18 years? See it.

P12 L267, stated “Out of the respondents….”. Please change in to “Out of the **total** respondents….”

P12 L269 put (.) after the word “water” and start with But….

However, “But 87(84.5%) of cases and 270 43(20.9%) of controls, did not treat drinking water at home.” Those figures are not present in the table. Why?

P13 L280, stated “poor hand washing practices”. What are your measurements and cut-off points to say poor or good? The same to that of “Knowledge of major risk factors” (Table 4)

P13 L 284-285, stated “… and 41(39.8%) of cases and 33(16%) 285 of controls did not receive vitamin-A supplementation”. The figures are not present in the table 4. Why?

**Discussion**

The discussion is very poor. It is based on results comparison. The discussion must include practical implications and “why” these results are relevant.

P18 L364, make the 2 references into 1.

P18 L398 stated “Therefore, awareness creation among mothers/ caretakers about the importance of action taken to halt acute diarrhea should be given attention.” To me, this is recommendation. Take it to its topic.

P18-19 L396- 398, states “Similarly, the odds of developing diarrheal morbidity were higher among children whose mothers/caretakers had better knowledge about the causes of diarrhea and had hand washing practice (AOR = 2.46, 95% CI: 1.07, 5.63) [23].” How it could be similar? This result is also contrary to facts.

**Conclusion**

The conclusion is good. However, it might be better than this.

P19 L426-427, stated “Providing simple and “easy to understand” information to the mothers/caretakers on major risk factors of acute diarrhea.” Unclear, modify this phrase.

**Availability of data**

The authors stated “The datasets used and/or analyzed during the current study are available in this manuscript. However, the corresponding author on reasonable request can submit the original dataset.”

Why not submitted without request during the submission time?

**References**

Some of the references used were too old. Moreover, see the reference style of the journal because some of these are not according the journal.
