# Supplementary material for: Behavioral and environmental determinants of acute diarrhea among under-five children from public health facilities of Siyadebirena Wayu district, north Shoa zone, Amhara regional state, Ethiopia: Unmatched case-control study
Source: PLoS One. 2021 Nov 22;16(11):e0259828. doi: 10.1371/journal.pone.0259828 (PMC8608321; doi:10.1371/journal.pone.0259828)
Supplement: Attachment — Submitted filename: Response to the reviewers.docx [file pone.0259828.s007.docx]

Response to the editor/reviewers

PONE-D-20-36640
Behavioral and Environmental Determinants of Acute Diarrhea among Under-five Children from Public Health Facilities of Siyadebirena Wayu District, North Shoa Zone, Amhara Regional State, Ethiopia: Unmatched Case-control Study
Dear Dr. Avanti Dey, Dr. G. Trueba, and Dr. Aderajew M, thank you very much for your comprehensive suggestions and comments. We are lucky to have you as an editor and reviewer. We have gone through each and every concern you have provided. Kindly addressed all the points raised and presented in the table below. Moreover, after taking all corrections based on reviewers (2) comments, we considered 1 – 10 editor(s) concerns too. Therefore, (1) we followed PLOS ONE style requirements, (2) we included supplementary information (e.g., S1 annex), (3) consents considered in method section, (4) data availability statement, (5) Funding statement, (6) Competing interests, (7) Figures 1 and 2 properly referenced in the text, (8) Figure 2 deleted since editorial error, (9) Table 1 referred, and (10) Ethics were included in method section. Finally, we attached 3 files, namely; response to the editor, manuscript with trach change, the revised version of the manuscript including the supporting materials (4).

| Points, questions, or suggestions | Responses or rebuttal or answers | Remarks |
| --- | --- | --- |
| **Reviewer #1**  (1) In the summary (and in other sections throughout the manuscript) the authors use the word “faces” instead of “feces” | We accepted and changed the word “faces” in to “feces”. Thank you | We have changed 7 words and checked all the document. |
| (2) Lines 40, in the summary the authors use two ways to express the odds ratio’s Cis. | Corrected. | We changed one “-“ to “,” |
| (3) The introduction section does not have a fluid narrative. The text should begin with a global status explanation of the diarrheal diseases (mortality and morbidity), followed by regional or developing countries and the Ethiopian situation. The text has redundant information (not only in the introduction). For example, the definition of diarrhea and its consequences are addressed in lines 58-60, 63-67, 71-75, 142-144; the risk factors allude in lines 60-62, 69-71, 81-83, 92-94; and the access to treatment in lines 86-87, 95-96. | Dear reviewers, as much as possible, we tried to revise the introduction section by avoiding redundancies, adding the aim of the study, and rewriting the sentences. The revised version of this paper contains the revision in track changes. | Thank you very much. |
| (4) Line 78. Very confusing sentence “….acute diarrheal diseases secondary to acute gastroenteritis” | Revised with thanks. |  |
| (5) There other factors which have been associated with acute diarrhea in Ethiopia, the paper should include these factors (with the respective references) in the introduction or discussion sections. | We tried to address factors associated with diarrhea in Ethiopia, Africa, and other nations in the world. |  |
| **(**6) Lines 76-77. Rewrite the sentence | Done. |  |
| **(**7) Lines 92-94: Rewrite the sentence | Done. |  |
| **(**8) Line 105: It is unclear why the authors describe the number of males in an Ethiopian region. | We rewrote the sentence. | Thank you. |
| **(**9) The aim of the study is in the abstract but not in the introduction. | We incorporated the aim of the study. |  |
| **(**10) Lines: 39, 106, 314-316, Some terms are local for the Ethiopian readers, however for an international audience they are confusing. For example, the ETB currency can be explained in poverty threshold in Euros or US dollars. The term “urban Kebele” should be explained. | We changed the word “Kebele” in to “smallest administrative unit” and the currency “ETB” into USD. | Thank you. |
| **(**11) Figure 1 is low quality | We tried to adjust the resolution of the figure. |  |
| **(**12) In lines 87-91 (also 96-99), the authors mention some Ethiopian studies about diarrhea prevalence. The authors must discuss in more detail the differences between settings, incomes, study design, the accomplishment of WHO recommendations, and other variables that explain such variances. | We revised considering the reviewers comments. | Thank you. |
| **(**13) Line 134, replace “was” with “were” | Corrected. |  |
| **(**14) Line 144, explain that controls came from children attending health centers | We explained how the controls were selected from the same health center and hospital. | Thank you so much. |
| **(**15) Line 197, it will be useful to have a supplemental document showing the questionnaire, it is important to see the questions that the authors used to assess the parents’ knowledge about risk factors. | English and Amharic version of the questionnaire is attached as a supplementary file. |  |
| **(**16) The authors need to explain the "institution-based" design. (line 118) | Out of four health facilities, this study included two health facilities. Thus, we tried to revise the expression. | Thank you. |
| **(**17) Legend of Table 3 should be rewritten | Done. |  |
| **(**18) Tables should be formatted following the guidelines of the journal and legends should be self-explanatory. The acronyms such as COR and AOR should be explained in the legend. | We added legend to the table 5 based on the suggestion. |  |
| **(**19) In table 5, statistically significant values seem to be written in bold. This should be indicated in the legend. | We explained significant variables by adding “*” and Note as a legend. |  |
| **(**20) The methodology section has redundancies in the text. First, the case definition is in lines 122-124, 131-132, 142-144 with slight differences that confused the readers. The same case is the definition of controls (lines 126-127, 144-145). Third, the dates are mentioned in lines 118-119, and 131. The estimation of the sample size is segmented within the text in lines 135-141 and 150-157. As mentioned, this segmentation of the information and redundancy confuse the readers. | Dear Dr. G. Trueba, as much possible we refined the paragraph to avoid redundancies. | Thank you very much. |
| **(**21) The contribution of the authors should be in the respective section, please check lines 205 and 219. | Corrected with thanks. |  |
| **(**22) The staff training is mentioned twice in 201-203 lines and 212-14 lines. | Training was incorporated under quality control section and removed from data collection tool section. |  |
| **(**23) The text needs to be more concise, please synthesize the methods section and avoid redundancies (lines 197-242). | We deleted some redundant information from the manuscript. |  |
| **(**24) The authors calculated a sample size of 315 participants however, they include only 309 (line 253). Explain the reasons for withdrawing the rest of the participants in the result section (it is slightly mentioned in lines 145-148). | One of the basic principles of Ethics in research is “autonomy” which is expressed in terms of consent of mothers/ caregivers. During consent, 6 study subjects refused to participate. |  |
| **(**25) The results section has several redundancies between the text and the tables. The tables need to be interpreted for the authors to highlight the important findings. As an example, the monthly income is mentioned in table 2, and in table 5 with the same data. Table 5 format is an improvement of Tables 2, 3, and 4 and permits the reader to get more information and engagement. The authors should use the format of Table 5 for all the tables because it includes the statistical analysis with all the other variables. | We edited table 2, 3, and 4 in the way that table 5 is formatted. Moreover, to avoid redundancies, tables 2, 3, and 4 are taken as a supplementary material. | Thank you very much. |
| **(**26) The discussion section should be rewritten to synthesize some ideas. The authors of this study use the statistical significance data (AOR) of other similar studies to compare their findings. | Some ideas like; handwashing practices, safe disposal of human excreta, community lead total sanitation, and eating food immediately after cooking were added. Moreover, some descriptions were revised. |  |
| **(**27) The study findings are suitable to propose recommendations to reduce the incidence of diarrheal diseases in this Ethiopian setting. The discussion needs a statement for recommendations, to gather all the ones disperse within the text (lines 337-339, 361-366, 376-379, 392-393, 421-430). The authors do not address the importance of public health policies and actions (and maybe the most supported) to improve sanitation and hygiene. | We included recommendations that promotes;   1. Handwashing practices with clean water and soap. 2. Safe disposal of human excreta – community lead total sanitation. 3. Eating foods immediately after cooking and avoid eating leftover foods. | Thank you very much. |
| **(**28) One of the main limitations of this study is that it does not consider causative agents and the pathogens involved in different factors may be different. For example, handwashing (331-339), drinking water contamination (347-349), no fresh food consumption (370-379), among others. This limitation has to be mentioned in the text. This approach can guide the recommendations for other studies. | Noted with thanks. Thus, we included this limitation and recommended accordingly. |  |
| **(**29) Lines 380-401 should be massively condensed. | We deleted some of the sentences and condensed the structure of the sentences too. |  |
| **(**30) Line 410 “Not” should be “not” | Done. |  |
| Reviewer #2  **Abstract section**  Page 2 line 33 “Methods: “Facility-based unmatched case-control study design was employed from March 12, 34 2019, to May 12, 2019.”  Better to be *“****Facility-based unmatched case-control study was conducted from March 12, 2019, to May 12, 2019.”*** | Amended as the suggestion. | Thank you. |
| Page 2 line 37 “The binary logistic regression model was employed to evaluate the independent effect of the predictor variables on acute childhood diarrhea”.  Better to be “***To analysis the data, binary logistic regression and multivariable logistic regression analysis was conducted***.” | We rewrote the sentence. |  |
| Introduction section |  |  |
| Pages (P)3, L 67-68; Page 3, line (L) 76-77; this part lacks essential references | Reference is inserted. |  |
| P3,L 58-60 which states “It is caused by bacterial, viral, and parasitic organisms and is usually a symptom of gastrointestinal infection **which can be caused by a variety of bacterial, viral, and parasitic organisms. Please change it into”** It is caused by bacterial, viral, and parasitic organisms and is usually causes gastrointestinal infection.” | Corrected with thanks. |  |
| P3 L62, please add the word “practice” after the word “sanitation” | Done. |  |
| P3 L82, delete the word “the” | Done. |  |
| P4 L88 add (,) after the word” revealed that” | Done. |  |
| Methodology  P8 L201, “Data was collected by a nurse who works at the under-five OPD/inpatient pediatrics ward of the health center and hospital.” Do you mean the data was collected by 1 nurse? Did you provided training for 1 data collector? I do not think so. See it. | Data was collected by 5 nurses and 2 supervisors managed the process. Thus, we corrected the expression. |  |
| P8 L208, delete the word “the” and replace it with “A” | Done. |  |
| Scientifically, in binary logistic regression analysis, a variable which has a P-value < 0.05 must be included in the multivariable analysis. | Yes indeed. We analyzed the data based on the reviewer concern. Thus, for bivariable logistic regression, to compute crude odds ratio we used p-value <0.2 and whereas in multivariable logistic regression, we used p-value <0.05. As the reviewer indicated we followed sound statistical cut-offs. |  |
| Result section  P10 L252 delete (’) which is located after the word “respondents.” | Done. |  |
| P10 L255 states “Forty-nine **(47.6 %)” which** is wrong. | The total number of cases were 103 and that of controls were 206. Thus, 49/103 gives 47.6%. Therefore, we corrected the writing. | Thank you. |
| P10 L 256 – 257, stated “Regarding the **age of children,** the average was 32.31 257 (±5.6) years for cases and 32.18 (±5.6) for controls. Can we call children whose age 32.3 and 32. 18 years? See it. | Sorry for the editorial errors. We edited as the mean (± SD) age of mothers was 32.31 (±5.6) years for cases and 32.18 (±5.6) for controls. |  |
| P12 L267, stated “Out of the respondents….”. Please change in to “Out of the **total** respondents….” | Done. |  |
| P12 L269 put (.) after the word “water” and start with But…. | Done. |  |
| However, “But 87(84.5%) of cases and 270 43(20.9%) of controls, did not treat drinking water at home.” Those figures are not present in the table. Why? | This sentence has editorial error and corrected as “But 86(83.5%) of cases and 56(27.2%) of controls, did not treat their drinking water at home”. |  |
| P13 L280, stated “poor hand washing practices”. What are your measurements and cut-off points to say poor or good? The same to that of “Knowledge of major risk factors” (Table 4) | **Good knowledge about major risk factors:** Respondents who mentioned at least three major risk factors such as none vaccinated for Rota virus and measles, lack of early initiation and exclusive breast feeding, using unsafe water for drinking and food preparation, lack of hand washing practice with soap at critical times, eating un properly handled food and lack of latrine facility (these options are available on the questionnaire).  **Poor knowledge about major risk factors:** Refers to respondents who mentioned at most two risk factors stated above.  **Good knowledge on action taken:** Respondents who mentioned at least three action taken such as take him/her to health institution, take him/her to traditional healer, increasing feeding, give him/her ORS, give him/her cereal based food, not stop/decrease feeding, homemade treatment (the options are on questionnaire).  **Poor knowledge on action taken:** Refers to respondents who mentioned at most two actions taken during acute diarrhea stated above. |  |
| P13 L 284-285, stated “… and 41(39.8%) of cases and 33(16%) of controls did not receive vitamin-A supplementation”. The figures are not present in the table 4. Why? | The figures are presented in table 5. Now available in table 4. |  |
| **Discussion section**  P18 L364, make the 2 references into 1. | Done. |  |
| P18 L398 stated “Therefore, awareness creation among mothers/ caretakers about the importance of action taken to halt acute diarrhea should be given attention.” To me, this is recommendation. | Incorporated to recommendation section. Moreover, we added other recommendations. |  |
| P18-19 L396- 398, states “Similarly, the odds of developing diarrheal morbidity were higher among children whose mothers/caretakers had better knowledge about the causes of diarrhea and had hand washing practice (AOR = 2.46, 95% CI: 1.07, 5.63) [23].” How it could be similar? This result is also contrary to facts. | We tried to rephrase the sentences to avoid confusion. |  |
| P19 L426-427, stated “Providing simple and “easy to understand” information to the mothers/caretakers on major risk factors of acute diarrhea.” Unclear, modify this phrase. | We revised this section by adding other ideas. |  |
| **Declaration section, Availability of data**  The authors stated “The datasets used and/or analyzed during the current study are available in this manuscript. However, the corresponding author on reasonable request can submit the original dataset.” Why not submitted without request during the submission time? | All relevant data are within the paper and its Supporting Information files. |  |
| **References**  Some of the references used were too old. Moreover, see the reference style of the journal because some of these are not according the journal. | Reference #15 is too old (1997) and it is used to address the cut-off points in bivariable and multivariable logistic regression. Moreover, the citation and bibliography used in this manuscript was based on “Plos manuscript submission guideline modified in April 2021” |  |

**Behailu Tariku Derseh- the corresponding author**
