# Supplementary material for: Behavioral and environmental determinants of acute diarrhea among under-five children from public health facilities of Siyadebirena Wayu district, north Shoa zone, Amhara regional state, Ethiopia: Unmatched case-control study
Source: PLoS One. 2021 Nov 22;16(11):e0259828. doi: 10.1371/journal.pone.0259828 (PMC8608321; doi:10.1371/journal.pone.0259828)
Supplement: Attachment — Submitted filename: Response to the editor V3.docx [file pone.0259828.s008.docx]

**Date 10/24/2021**

**Response to the editor**

**PONE-D-20-36640R1**

Behavioral and environmental determinants of acute diarrhea among under-five children from public health facilities of Siyadebirena Wayu district, north shoa zone, amhara regional state, Ethiopia: unmatched case-control study

Current status: **Minor revision**.

Dear Dr. Gabriel Trueba, thank you very much for showing us addressing Plos one journal requirements. In the second version of the manuscript we addressed all points raised by the reviewers and editor. Now, we have checked all the references, make figure compatible with Plos requirement (PACE), and proof read the manuscript. Since the beginning of our submission, we learnt a lot and lucky to send the manuscript to PLOS ONE!

Kindly addressed all the points raised by you and presented in the table below. Moreover, after corrections, we attached 3 files, namely; response to the editor, manuscript with trach change, the revised version of the manuscript including the supporting materials.

| Points, questions, or suggestions | Responses or rebuttal or answers | Remarks |
| --- | --- | --- |
| **Journal Requirements:**  Please review your reference list to ensure that it is complete and correct. If you have cited papers that have been retracted, please include the rationale for doing so in the manuscript text, or remove these references and replace them with relevant current references. Any changes to the reference list should be mentioned in the rebuttal letter that accompanies your revised manuscript. If you need to cite a retracted article, indicate the article’s retracted status in the References list and also include a citation and full reference for the retraction notice. | Reference number 11 and 17 are similar but cited as two different articles. With many thanks, we revised this section. Thus, previously the # articles cited were 33, now become 32. | We used Mendeley Desktop referencing software. |
| **Additional Editor Comments (if provided):** Page 2 lines 42 and 46 Replace the sentence "consuming a child with leftover food" with "children consuming left-over food stored at room temperature" | Accepted and corrected accordingly with thanks. |  |
| **Reviewers' comments:**  [NOTE: If reviewer comments were submitted as an attachment file, they will be attached to this email and accessible via the submission site. Please log into your account, locate the manuscript record, and check for the action link "View Attachments". If this link does not appear, there are no attachment files.] | No attachment at all, however, we tried to address all the points in the second version of the manuscript. | Thank you. |
| While revising your submission, please upload your figure files to the Preflight Analysis and Conversion Engine (PACE) digital diagnostic tool, <https://pacev2.apexcovantage.com/>. PACE helps ensure that figures meet PLOS requirements. To use PACE, you must first register as a user. Registration is free. Then, login and navigate to the UPLOAD tab, where you will find detailed instructions on how to use the tool. If you encounter any issues or have any questions when using PACE, please email PLOS at [figures@plos.org](mailto:figures@plos.org). Please note that Supporting Information files do not need this step. | Done with thanks. Figure 1 is changed into Preflight Analysis and Conversion Engine file (PACE). |  |

**With regards**

**The corresponding author**
